# Unveiling a Novel Zearalenone Biodegradation Pathway in *Metarhizium anisopliae* and Elucidating the Role of Cytochrome P450

**DOI:** 10.3390/ijms26062547

**Published:** 2025-03-12

**Authors:** Monika Nowak, Elżbieta Kozłowska, Justyna Agier, Aleksandra Góralczyk-Bińkowska, Sylwia Różalska

**Affiliations:** 1 Department of Industrial Microbiology and Biotechnology, Faculty of Biology and Environmental Protection, University of Lodz, 90-237 Lodz, Poland; monika.nowak@biol.uni.lodz.pl; 2 Centre of Molecular Studies on Civilization Diseases MOLecoLAB, Department of Microbiology, Genetics and Experimental Immunology, Medical University of Lodz, 92-215 Lodz, Poland; elzbieta.kozlowska@umed.lodz.pl (E.K.); justyna.agier@umed.lodz.pl (J.A.); aleksandra.goralczyk-binkowska@umed.lodz.pl (A.G.-B.)

**Keywords:** fungal detoxification, bioremediation, zearalenone, mycotoxins, UHPLC-HRMS, cytochrome P450

## Abstract

*Metarhizium* fungi, essential for ecosystem function and commonly utilised in pest control, often occupy ecological niches contaminated by toxic compounds of both anthropogenic and microbiological origin. The present study reveals the potential of *Metarhizium anisopliae* for biodegradation of the *Fusarium* mycotoxin zearalenone (ZEN), a common contaminant of crops that poses a significant threat to human and animal health due to its oestrogenic potential and toxicity. A key aspect of the pathway described is the degradation of ZEN by cleaving the lactone bond, which results in a significant reduction in mycotoxin toxicity, highlighting the fungus’s bioremediation potential. Furthermore, this study provides the first evidence of subsequent degradation of ZEN metabolites through progressive shortening of the aliphatic chain, primarily via alternating oxidation and demethylation, ultimately yielding trihydroxybenzene. Significantly, lactone bond cleavage occurred not only in ZEN itself but also in its reduced forms, the zearalanols, formed through the initial reduction of ZEN to zearalenols. Elevated mRNA levels of cytochrome P450 (CYP450) monooxygenases in *M. anisopliae* exposed to ZEN indicate their significant involvement in degradation mechanisms. Intriguingly, the inhibition of CYP450 activity resulted in a substantial shift in the quantitative ratio of α- and β-epimers of zearalenols and zearalanols. The observed alteration towards β-form production likely stems from the inhibition of other CYP450-dependent reactions, indirectly influencing ZEN reduction pathways—a particularly noteworthy finding. These insights are crucial for developing strategies to utilise *M. anisopliae* in the bioremediation of ZEN-contaminated areas.

## 1. Introduction

*Metarhizium* fungi, as soil-dwelling Ascomycetes, fulfil various ecological roles, including acting as saprotrophs, plant symbionts, and insect pathogens. These microorganisms are crucial in a wide range of trophic interactions, significantly contributing to essential ecosystem functions [1]. Moreover, *Metarhizium* species are extensively applied as fungal biocontrol agents for managing crop pest populations [2]. These fungi thrive in diverse ecological niches, encountering toxic compounds such as heavy metals and pesticides. Through their adaptable metabolic processes, they are able to biotransform or degrade xenobiotics [3,4,5,6,7].

Nevertheless, the ecological niches inhabited by *Metarhizium* may also host other fungal species proficient in producing various toxic metabolites. Zearalenone (ZEN) is a well-known mycotoxin produced by several *Fusarium* species that commonly contaminate crops. This poses a substantial risk to both human and animal health, owing to its oestrogenic properties and inherent toxicity [8,9]. In both humans and animals, ZEN undergoes biotransformation via two primary phases. Phase I metabolism, encompassing oxidation, reduction, and hydrolysis, acts directly on the parent ZEN molecule. Phase II metabolism follows, involving conjugation reactions that further modify the ZEN metabolites. In vertebrates, ZEN metabolism exhibits species-specific variations. While reduction represents the dominant metabolic pathway in pigs and humans, hydroxylation prevails in species such as rats, chickens, goats, and cows. Notably, rats and chickens exhibit a more diverse metabolic profile, featuring reduction, hydroxylation, and glucuronidation as major pathways, with sulfation playing a significant role in chickens [10]. The biotransformation of ZEN involves a complex interplay of enzymatic reactions, primarily driven by hydroxysteroid dehydrogenases (3α- and 3β-HSD) and cytochrome P450 enzymes (CYP450), leading to the formation of diverse hydroxylated metabolites [11,12], such as α/β-zearalenol and α/β-zearalanol.

Although the general pathways of zearalenone biotransformation in humans and animals are well-characterised, the specific mechanisms employed by environmentally relevant fungi, particularly those coexisting with *Fusarium* in nature, remain largely unexploited [13,14].

While several soil-borne fungal species have showcased their capacity to biotransform zearalenone into derivatives, such as α/β-zearalenol and α/β-zearalanol, and also form conjugates by adding sulphate, glucose, or glucuronide [15,16], *Clonostachys rosea* IFO 7063 remained unique in its ability to degrade ZEN by hydrolysing the lactone ring [17]. This enzymatic cleavage of the lactone structure is a key distinction, as the other biotransformations referenced do not interfere with the integrity of the lactone ring [14,18,19].

*Metarhizium* is a genus of fungi that inhabits diverse soil environments. It plays numerous ecological roles, and this lifestyle requires a versatile and diverse metabolism [2]. Cytochrome P450s are key enzymes contributing to this metabolic versatility, enabling adaptation to specific niches. Notably, CYP52 and CYP53 families are involved in xenobiotic metabolism and/or detoxification [20]. For example, *Metarhizium* fungi possess a highly active CYP450, whose role in the degradation of insect epicuticular hydrocarbons and toxic contaminant nonylphenol has been previously confirmed [3,21].

Understanding the mechanisms and pathways through which *Metarhizium* species can transform or degrade ZEN could have important implications for mitigating the impact of this mycotoxin on agricultural products and the environment. This study investigates the biodegradation of ZEN by *Metarhizium anisopliae*, exploring its potential as a biocontrol agent to mitigate mycotoxin contamination in agriculture. Also, it provides novel insights into ZEN metabolism, highlighting the involvement of cytochrome P450 in the formation of critical metabolites. Our findings could contribute to developing sustainable and eco-friendly strategies for mitigating the harmful effects of ZEN and other mycotoxins.

## 2. Results

### 2.1. Zearalenone Biodegradation Pathway

*Metarhizium anisopliae* ARSEF7487 eliminated *Fusarium* mycotoxin zearalenone (ZEN) from the growth environment. *M. anisopliae* removed approximately 90.8% of ZEN from the culture medium during a 14-day period. After 2 days of incubation, an approximately 50% loss of ZEN was observed. A further substantial decrease in ZEN concentration, approximately 17%, was observed between days 4 and 5, resulting in an overall elimination rate of 80% after 5 days of incubation [19] (Table 1).

**Figure 1:**
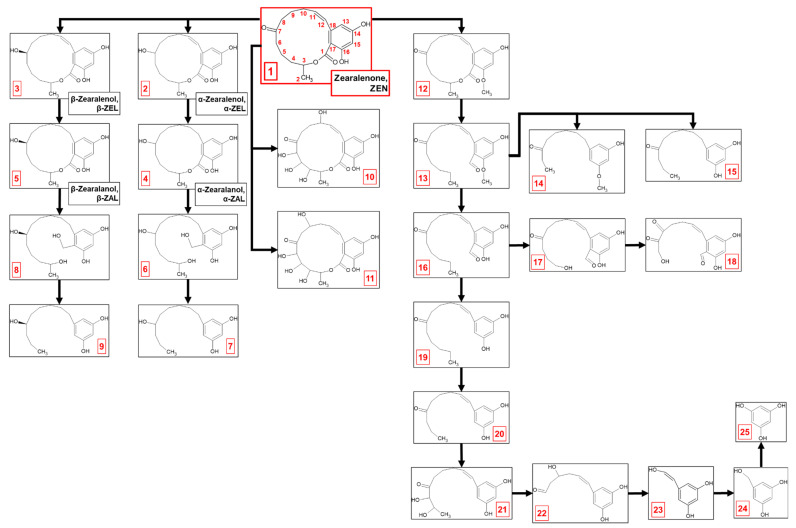
Zearalenone biodegradation by *M. anisopliae*—proposition of pathway. Metabolite numbers correspond to the designated identifiers in Table 2.

The process of ZEN elimination appears to occur via a complex, multi-step metabolic pathway involving both biodegradation and biotransformation (Figure 1, Table 2). Within the first 2 days of cultivation of *M. anisopliae* with ZEN (compound **1**; 317.1387 *m/z*), two primary derivatives, α-zearalenol (metabolite **2**; 319.1545 *m/z*) and β-zearalenol (metabolite **3**; 319.1549 *m/z*), were formed. These stereoisomers result from the reduction in ZEN at the C7-ketonic carbonyl group within the lactone ring. Further reduction in zearalenols at the C11-C12 double bond within the lactone ring led to the formation of α-zearalanol (metabolite **4**; 321.1697 *m/z*) and β-zearalanol (metabolite **5**; 321.1696 *m/z*).

By the fourth day of incubation, two metabolites presumed to be formed through the tetraoxidation of ZEN were identified. Detailed analysis of the mass spectra suggested that both metabolite **10** (381.1179 *m/z*) and metabolite **11** (381.118 *m/z*) underwent triple oxidation within the lactone ring, which occurred between the methyl group at C2 and the ketone group at C7. These two metabolites differed in the location of a fourth hydroxyl group, which was attached at C10 and C9 in metabolites **10** and **11**, respectively. Beyond identifying ZEN oxidation products, further metabolism of zearalanols was confirmed by the identification of metabolites **7** (251.1644 *m/z*) and **9** (251.1641 *m/z*). These transformations probably involved cleavage of the lactone ring by hydrolysis, followed by demethylation, dehydroxylation, and removal of the ketone group.

The process of ZEN elimination intensified after 5 days of cultivation, and a more complex metabolic profile was revealed. Biodegradation products became the dominant forms detected, with biotransformation products present to a lesser extent. Intermediate metabolites between zearalanols and derivatives **7** and **9** were observed. Metabolite **6** (325.2006 *m/z*) and metabolite **8** (325.2009 *m/z*) showed that lactone ring cleavage occurred via a multi-step process. The lactone bond likely underwent hydrolysis, causing the ring to open and form a hydroxyl group (-OH) at C3 and a carboxyl group (-COOH) at C1. Subsequently, the ketone group at C1 was reduced from C=O to C-OH, resulting in a geminal diol (two hydroxyl groups) at C1. Finally, dehydroxylation then removed one of these hydroxyl groups, leading to the formation of metabolite **6** from α-zearalanol degradation and metabolite **8** from β-zearalanol degradation.

In addition to biotransformation via ketone group reduction at C7, double bond reduction at C11-C12 within the lactone ring, or tetraoxidation, a key branching point in the ZEN degradation pathway involves methylation at C16 in the aromatic ring, yielding metabolite **12** (331.1541 *m/z*). This methylation step seemed to prime ZEN for further breakdown. Subsequent hydrolysis of the lactone ring and dehydroxylation at both C1 and C3 in metabolite **12** yielded metabolite **13** (317.1747 *m/z*).

Metabolite **13** then underwent a series of modifications, leading to the formation of three different derivatives. Metabolite **14** (249.1485 *m/z*) was formed through a series of modifications, including shortening of the aliphatic chain at the aromatic ring, reduction in the C11-C12 double bond, and demethylation, which further shortened the aliphatic chain towards the ketone group at C7. Metabolite **15** (249.149 *m/z*) was formed through a similar pathway to metabolite **14**, involving shortening of the aliphatic chain at the aromatic ring and reduction in the C11-C12 double bond. However, instead of a third demethylation on the aliphatic chain towards the ketone group at C7, demethylation at C16 in the aromatic ring was determined.

In parallel with the previous transformations, metabolite **13** underwent demethylation at C16 in the aromatic ring after lactone ring hydrolysis, yielding metabolite **16** (303.159 *m/z*). Metabolite **16** underwent further demethylation of the aliphatic chain towards the ketone group at C7, accompanied by the formation of a hydroxyl group at C4, yielding metabolite **17** (291.1228 *m/z*). Subsequent demethylation at C6 resulted in a hydroxyl group shift from C4 to C6. Oxidation at C8 then introduced a carbonyl group (C=O), forming metabolite **18** (277.0715 *m/z*).

Metabolite **16** also branched into an alternative pathway: reduction in the ketone group at C1 and subsequent demethylation produced metabolite **19** (275.1652 *m/z*), followed by demethylation-driven aliphatic chain shortening towards the ketone group at C7, which produced metabolite **20** (247.1331 *m/z*). Double oxidation at C6 and C5 yielded a metabolite **21** (279.1226 *m/z*). Metabolite **22** (221.0817 *m/z*) was formed by shortening the aliphatic chain to a ketone group at C7, followed by oxidation at C9, introducing a hydroxyl group. Metabolite **23** (151.0397 *m/z*) was formed through reduction in the ketone group and further degradation of the aliphatic chain towards the aromatic ring and introduced a hydroxyl group at C11. Reduction in the C11-C12 double bond and further demethylation resulted in forming metabolite **24** (139.0396 *m/z*). Complete degradation of the aliphatic chain, initiated by lactone ring cleavage, yielded metabolite **25** (125.0245 *m/z*), identified as trihydroxybenzene.

### 2.2. Cytochrome P450 Involvement in ZEN Biodegradation

#### 2.2.1. Cytochrome P450 Gene Expression Profiles in *M. anisopliae* During Zearalenone Degradation

**Figure 2:**
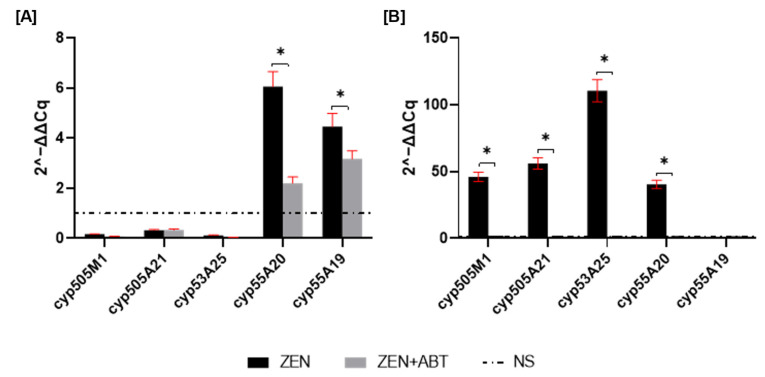
mRNA expression of genes involved in ZEN removal after 1 (**A**) and 2 (**B**) days of incubation. Results are presented as the mean ± SD. Statistically significant differences were marked as *—(*p* < 0.0001).

To assess changes in gene expression, qRT-PCR was performed to measure the fold change in cyp450 mRNA after 1 and 2 days of incubation. As shown in Figure 2A, *cyp55A20* and *cyp55A19* showed the highest mRNA expression levels. Supplementing ZEN cultures with the ABT inhibitor resulted in a 63% decrease in cyp55A20 and a 28% decrease in cyp55A19 mRNA expression. Transcription level changes for the remaining genes were insignificant at 1 day of incubation. However, by day 2 of incubation, most of the tested cyp450 transcripts (except the *cyp55A19* gene) showed significant overexpression, with fold changes greater than 40 (Figure 2B). The most prominent change was observed for *cyp55A25* (a gene encoding CYP450 monooxygenase), which exhibited a one-hundred-and-ten-fold increase in expression. Notably, treatment with the CYP450 inhibitor ABT resulted in a 97% reduction in the expression of all tested cyp450 transcripts in ZEN-treated cultures.

#### 2.2.2. Changes in the Elimination Rate of Zearalenone and the Formation of Hydroxylated Derivatives After Inhibitory Assay with 1-Aminobenzotriazole

**Figure 3:**
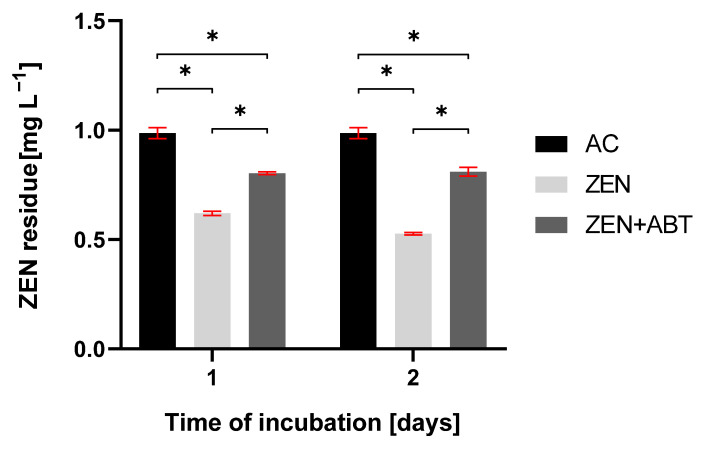
The residue of zearalenone (ZEN) in abiotic control (AC), tested sample of *Metarhizium anisopliae* treated with ZEN, and tested sample of *Metarhizium anisopliae* treated with ZEN and 1-aminobenzotriazole (ZEN + ABT) after 1 and 2 days of incubation. Results are presented as the mean ± SD. Statistically significant differences were marked as *—(*p* < 0.0001).

As depicted in Table 1, *M. anisopliae* effectively removed ZEN from the growth medium, reaching 50% of mycotoxin loss after 2 days of incubation. Supplementation with a CYP450 inhibitor, 1-aminobenzotriazole (ABT), resulted in a statistically significant, although incomplete, inhibition of the ZEN removal on 1 and 2 days of incubation (Figure 3). In these ABT-treated samples, ZEN concentration remained constant at approximately 0.8 mg L^−1^.

**Figure 4:**
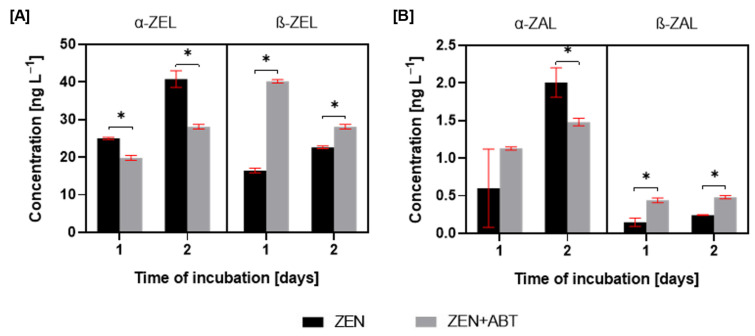
The concentration of α-zearalenol (α-ZEL) and β-zearalenol (β-ZEL) (**A**) and concentration of α-zearalanol (α-ZEL) and β-zearalanol (β-ZEL) (**B**) in tested samples of Metarhizium anisopliae treated with zearalenone (ZEN) or zearalenone with 1-aminobenzotriazole (ZEN + ABT) after 1 and 2 days of incubation. Results are presented as the mean ± SD. Statistically significant differences were marked as *—(*p* < 0.01).

The incubation of *M. anisopliae* with zearalenone resulted in the formation of four primary metabolites: α-zearalenol (α-ZEL), β-zearalenol (β-ZEL), α-zearalanol (α-ZAL), and β-zearalanol (β-ZAL) (Figure 1). The supplementation of ZEN cultures with the CYP450 inhibitor, ABT, resulted in a decrease in α-ZEL production and a simultaneous increase in β-ZEL (Figure 4). For the ZEN-treated cultures, the ratio of α-ZEL to β-ZEL changed from 1.5:1 to 1:2.4 after CYP450 inhibition at 1 day of incubation and from 1.8:1 to 1:1.2 at 2 days of incubation.

In contrast to the observed trends in ZEL metabolites, the ZAL analysis revealed distinct patterns. Still, the α-form was predominant (Figure 4). Although no statistically significant differences in α-ZAL production were observed between ZEN and ZEN + ABT treatments after 1 day, a significant inhibition of α-ZAL production was evident in the presence of the inhibitor after 2 days. While β-ZAL production was relatively low in ZEN-treated cultures, adding a CYP450 inhibitor resulted in a two-fold increase in β-ZAL levels at 1 and 2 days of incubation.

## 3. Discussion

Zearalenone, a mycotoxin produced by species of the *Fusarium* genus, is widely recognised for contaminating staple crops, causing considerable economic losses within the food and livestock industries. Its significant oestrogenic properties lead to detrimental effects on human and animal health, including reproductive disorders, disruption of endocrine function, genotoxicity, and potential carcinogenicity [9,22]. Comprehending the potential microbial-mediated transformations of zearalenone in the environment is imperative, as these processes could generate metabolites with heightened toxicity or estrogenicity, thereby presenting a greater risk than the parent compound. Additionally, the emergence of ‘masked mycotoxins’, while seemingly detoxified, raises concerns regarding their potential to revert to their original toxic form [16,23,24]. Entomopathogenic fungi of the genus *Metarhizium* are ubiquitous in soil ecosystems and play a crucial role in agriculture, as their use as biopesticides offers a sustainable alternative to chemical insecticides [25]. Beyond their insecticidal properties, *Metarhizium* species demonstrate a remarkable ability to remediate environmental contaminants [3,4,5,6,7], including zearalenone [19]. Our previous studies have shown that *M. anisopliae* can effectively remove ZEN, both when present as a pure substance and as a native component of a complex mixture of metabolites from *Fusarium graminearum.* This process occurs through biotransformation, specifically by reduction to zearalenols and zearalanols [19], which represent the most prevalent transformation products of this mycotoxin [26], also observed in fungi [18,27]. Compared to the total amount of mycotoxin eliminated, the relatively low contents of zearalenols and zearalanols detected during ZEN biotransformation by *M. anisopliae* suggested that ZEN underwent a further transformation. The current investigation illustrates that *M. anisopliae* effectively degraded ZEN through a multi-step metabolic pathway. The degradation process was initiated with methylation and lactone bond cleavage and followed by complete degradation of the aliphatic chain, ultimately yielding trihydroxybenzene. The case we present regarding the biodegradation of ZEN by *M. anisopliae,* achieved through the cleavage of the lactone bond, constitutes a noteworthy contribution to the existing literature. To the best of our knowledge, only one prior study has documented a fungus demonstrating such cleavage capabilities, whereas the majority of research conducted has predominantly centred on biotransformation rather than complete degradation of this compound mycotoxin.

The biotransformation of zearalenone predominantly encompasses reduction, oxidation, or conjugation with molecules such as glucose, glucuronic acid, or sulphates. For example, species within the genus *Cordyceps* primarily biotransform zearalenone through sulfonation and glucuronidation, followed by oxidation. However, the biotransformation pathway includes derivatives formed via reduction, oxidation, and glycosylation [18]. *Rhizopus* fungi, however, employ glucose conjugation at various carbon positions within the ZEN molecule [28]. Both *Aspergillus oryzae* and *Sphaerodes mycoparasitica* form ZEN conjugates with a sulphate group [28,29]. *M. anisopliae* transforms ZEN through a distinct pathway. Unlike many reported cases, no glucose or sulphate conjugates were observed. While it follows the common reductive route, converting ZEN to zearalenols and zearalanols, its oxidative process is unique. Unlike *Cordyceps* strains, which typically introduce one or two oxygen atoms [18], *M. anisopliae* carries out a tetra-oxidation.

Degradation of zearalenone via lactone bond cleavage is significantly less common. Kakeya et al. [17] reported a notable exception—the fungus *Clonostachys rosea* IFO 7063 efficiently degraded ZEN through lactone hydrolysis. This process yielded two primary metabolites: one with a carboxyl group at C1 and a hydroxyl group at C3 and a second resulting from carboxyl group removal. To our knowledge, this is the only report of the cleavage of a lactone bond in a ZEN molecule by a fungus. The *M. anisopliae* strain studied in this paper cleaved the lactone bond in ZEN in a quite similar way to *C. rosea.* Methylation of ZEN at C16 on the aromatic ring was followed by hydrolysis of the lactone ring and subsequent dehydroxylation at both C1 and C3. What distinguishes the ZEN biodegradation pathway by *M. anisopliae* is the following degradation of the molecule by shortening its aliphatic chain. This process is primarily facilitated through a mechanism of alternating oxidation and demethylation. Similarly to its degradation of ZEN, *M. anisopliae* degraded 4-*n*-nonylphenol through a comparable mechanism involving oxidation and demethylation, ultimately mineralising the xenobiotic to CO_2_ and H_2_O [3]. Furthermore, lactone bond cleavage was observed not only in ZEN itself during biodegradation by *M. anisopliae* but also in its reduced derivatives. ZEN reduction to zearalenols occurred first, followed by conversion to zearalanols, ultimately leading to lactone bond cleavage. This reaction is the first of its kind observed in fungi.

The literature consistently demonstrates the crucial role of cytochrome P450 monooxygenases in the elimination of toxic compounds by entomopathogenic fungi of the genus *Metarhizium*. For instance, inhibiting CYP450 activity in *Metarhizium robertsii* IM 6519, using the non-selective inhibitor proadifen, significantly hindered atrazine degradation and led to a decrease in the production of 2-hydroxy-atrazine and its subsequent metabolites [5]. Similarly, CYP450 inhibition in *M. robertsii* resulted in a four-fold reduction in the dibutyltin degradation rate, with a complete absence of the hydroxylated monobutyltin metabolite [7]. Furthermore, CYP450 monooxygenases played a critical role in the degradation of 4-*n*-nonylphenol by eight different *Metarhizium* fungal strains [3]. The presence of xenobiotic 4-*n*-nonylphenol in fungal cultures significantly increased CYP450 activity in microsomes. Additionally, treatment with the CYP450 inhibitor 1-aminobenzotriazole completely abolished the formation of hydroxylated derivatives of xenobiotic. Likewise, *M. anisopliae* exhibited significantly elevated CYP450 monooxygenase and cytochrome P450 reductase activity in the presence of zearalenone (except *cyp55A19* on the second day of incubation) (Figure 2B). The over forty-fold increase in mRNA expression of the four CYP450 genes strongly suggests their significant role in ZEN elimination. To date, the involvement of fungal CYP450 in ZEN elimination has only been described for *Cordyceps fumosorosea*, where CYP450 was involved in ZEN oxidation reactions [18]. Additional detailed information pertaining to the function of human CYP450 enzymes, specifically CYP1A2 and CYP3A4, in the transformation of ZEN is available. These isoforms have been identified as playing a role in the formation of ZEN metabolites characterised by the presence of a hydroxyl group [30]. In this study, inhibiting the activity of CYP450 with the non-selective inhibitor 1-aminobenzotriazole resulted in a ~20% reduction in ZEN elimination by *M. anisopliae* after one day of incubation. Still, it did not lead to complete inhibition of this process. Interestingly, CYP450 inhibition triggered a shift in the ZEN biotransformation: increased production of β-zearalenols and β-zearalanols, coupled with decreased formation of their α-isomers. Given the near-complete suppression of CYP450 genes expression through 1-aminobenzotriazole application, a direct involvement of these enzymes in the observed biotransformation is unlikely. Instead, the observed shift towards β-form production likely stems from inhibiting other CYP450-dependent reactions, indirectly influencing ZEN reduction pathways. This phenomenon, which has not been previously documented in the literature, warrants further investigation.

## 4. Materials and Methods

### 4.1. Fungal Strain and Chemicals

*Metarhizium anisopliae* ARSEF7487 was obtained from the ARSEF collection (The Agricultural Research Service Collection of Entomopathogenic Fungal Cultures, USDA-ARS, Ithaca, NY, USA). Zearalenone (ZEN), α/β-zearalenol (α/β-ZEL), and α/β-zearalanol (α/β-ZAL) were purchased from Cayman Chemical (Ann Arbor, MI, USA) (purity ≥ 98%). 1-Aminobenzotriazole (ABT) was purchased from Sigma-Aldrich (Steinheim, Germany). Stock solutions of ZEN, α/β-ZEL, and α/β-ZAL at the concentration of 1 mg mL^−1^ were prepared in methanol (Avantor Performance Materials, Gliwice, Poland), and a stock solution of ABT at the concentration of 100 mM was prepared in ethanol (Avantor Performance Materials, Gliwice, Poland). All stock solutions were prepared directly before the experiment. Reagents and solvents for liquid chromatography and mass spectrometry, as well as reagents not listed above, were purchased from Merck (Steinheim, Germany) unless otherwise stated.

### 4.2. Biodegradation Experiment

*Metarhizium anisopliae* ARSEF7487 was cultivated on ZT agar slants (glucose (4 g L^−1^), Difco yeast extract (4 g L^−1^), malt extract (6 °Blg), and agar (25 g L^−1^); pH 7) for 14 days and then was inoculated in Sabouraud Dextrose Broth (BioMaxima, Lublin, Poland) at a ratio of 1:9 and incubated on a rotary shaker (120 rpm) at 28 °C in the dark. After 1 day, 2 mL of the preculture was transferred to 18 mL of Czapek Dox Broth (BD-Difco, Bordeaux, France) in 100 mL Erlenmeyer flasks, and the fungal cultures were incubated under the same conditions. To determine the biodegradation products of ZEN, the following experimental setups were prepared: biotic controls of *M. anisopliae* without ZEN and tested cultures of *M. anisopliae* with the addition of ZEN (1 mg L^−1^). These setups were incubated for 2 days (when approximately 50% loss of ZEN was observed from the start of incubation) and 4 and 5 days (when approximately 17% loss of ZEN was observed between these time points) [19].

### 4.3. Sample Extraction and Preparation

Whole fungal cultures of *M. anisopliae* were extracted using the modified QuEChERS extraction procedure described previously [18,19]. Briefly, 10 mL of acetonitrile was added to 20 mL of the entire fungal culture and vortexed at 3000 rpm for 1 min. Next, QuEChERS salts (4 g MgSO_4_; 1 g NaCl; 1 g C_6_H_5_ Na_3_O_7_∙2H_2_O; 0.5 g C_6_H_6_Na_2_O_7_∙1.5 H_2_O) were added to each sample, and the samples were vortexed at 3000 rpm for the next minute and then centrifuged at 8000 rpm for 10 min. Directly after centrifugation, 7 mL of the top layer was evaporated to dryness under a pressure of 5 hPa at 40 °C. After evaporation, the residue was dissolved in 1 mL of methanol and then diluted ten-fold in water (LC-MS grade) for qualitative analysis of ZEN biodegradation products.

### 4.4. Cytochrome P450 Activity

#### 4.4.1. Cytochrome P450 Inhibitor Assay

*Metarhizium anisopliae* ARSEF7487 was cultivated on ZT agar slants for 14 days and then inoculated in Sabouraud Dextrose Broth at a ratio of 1:9 and incubated on a rotary shaker (120 rpm) at 28 °C in the dark. After 1 day, 2 mL of the preculture was transferred to 18 mL of Czapek Dox Broth in 100 mL Erlenmeyer flasks, and the fungal cultures were incubated under the same conditions. To determine the ZEN elimination rate and the formation of α/β-ZEL and α/β-ZAL after CYPP450 inhibition, the following experimental setups were prepared: biotic controls of *M. anisopliae* without any additives, tested cultures of *M. anisopliae* with the addition of ZEN (1 mg L^−1^), and tested cultures of *M. anisopliae* with the addition of ZEN (1 mg L^−1^) and ABT (1 mM) as a non-specific CYP450 inhibitor. These setups were incubated for 1 and 2 days, during which significant ZEN loss and high amounts of α/β-ZEL and α/β-ZAL were observed [19]. After incubation, the QuEChERS extraction procedure (described in Section 4.3) was performed. For the quantitative analysis of ZEN, 1 mL of the top layer was collected and then diluted ten-fold with LC-MS grade water. For the quantitative analysis of α/β-ZEL and α/β-ZAL, 7 mL of the top layer was evaporated to dryness under a pressure of 5 hPa at 40 °C. The residue was dissolved in 1 mL of methanol and then diluted ten-fold with LC-MS grade water.

#### 4.4.2. RNA Extraction, cDNA Synthesis and Quantitative RT-PCR (RT-qPCR)

Total RNA was extracted from 100 mg of wet biomass of *M. anisopliae* ARSEF7487 using TRI Reagent^®^ (Sigma-Aldrich, Steinheim, Germany). The fungus was cultivated for 1 or 2 days in three different experimental setups: biotic controls of *M. anisopliae* without any additives, tested cultures of *M. anisopliae* with the addition of ZEN (1 mg L^−1^), and tested cultures of *M. anisopliae* with the addition of ZEN (1 mg L^−1^) and ABT (1 mM). The RNA concentration (A260) and the purity (A260/A280) were measured with the BioPhotometer Plus UV/Vis (Eppendorf^®^, Hamburg, Germany). RNA samples were immediately frozen at −80 °C and stored until laboratory analysis. According to the manufacturer’s protocol, complementary DNA (cDNA) was synthesised using a High-Capacity cDNA Reverse Transcription Kit (Applied Biosystems, Foster City, CA, USA). Applied primers were previously designed in Primer3Web software v. 4.1.0 (Tartu, Estonia) and then confirmed via Primer-BLAST. The reference gene was selected based on Fang and Bidochka [31]. The sequences of primers are shown in Table 3. Gene expression was measured in triplicates using SsoAdvanced™ Universal SYBR^®^ Green Supermix (Bio-Rad Laboratories, Hercules, CA, USA) and Rotor-Gene Q (Qiagen, Hilden, Germany). The reaction mixture contained 2 μL of forward and reverse primers (Genomed, Warsaw, Poland), 1 μL of cDNA template, 5 μL of SsoAdvanced™ Universal SYBR^®^ Green Supermix, and 2 μL of PCR-grade water in a final volume of 10 μL. The PCR cycle conditions were as follows: 95 °C for 30 s, followed by 40 cycles of denaturation at 95 °C for 10 s, and then annealing/extension at 60 °C for 10 s. The fold changes in the tested samples were calculated based on the ΔΔCt method. The expression of mRNA transcripts was normalised to the ubi endogenous gene.

### 4.5. Analytical Methods

#### 4.5.1. Quantitative Analysis of Zearalenone

The analysis was conducted according to the method described by Nowak et al. [18,19] using an Agilent 1200 LC system (Agilent, Santa Clara, CA, USA) coupled with a QTRAP 4500 mass spectrometer (SCIEX, Framingham, MA, USA). Chromatographic separation was achieved on a Kinetex C18 column (50 × 2.1 mm, 5 μm; Phenomenex, Torrance, CA, USA) at 40 °C. The mobile phase was water (A) and methanol (B), both with 5 mM ammonium formate, at a flow rate of 0.5 mL min^−1^. The injection volume was 5 µL. The gradient started with 95% A for 0.25 min, then changed to 95% B from 0.25 to 1.5 min, held at 95% B for 2.5 min, and returned to the initial conditions between 4 and 4.1 min, maintaining until 6.0 min for equilibration.

Tandem mass spectral (MS-MS) analysis was accomplished with an electrospray ion source (ESI) in negative ionisation mode using multiple reaction monitoring (MRM). The ESI settings were as follows: curtain gas (CUR) at 25, Ion Spray Voltage (IS) at −4.5 kV, temperature (TEMP) at 550 °C, ion source gas 1 (GS1) at 40, and ion source gas 2 (GS2) at 60. The MRM transitions were *m*/*z* 317 → 131 (quantifier) and 317 → 175 (qualifier). The standard curve was linear from 25 to 1000 ng mL^−1^ (r = 0.9996).

#### 4.5.2. Quantitative Analysis of α/β-Zearalenol and α/β-Zearalanol

The analysis was conducted according to the method described by Nowak et al. [18,19] using an Agilent 1200 LC system (Agilent, Santa Clara, CA, USA) coupled with a QTRAP 4500 mass spectrometer (SCIEX, Framingham, MA, USA). Chromatographic separation was achieved on a Luna C8 column (150 × 2 mm, particle size: 5 μm; Phenomenex, Torrance, CA, USA) at 40 °C. The mobile phase was water (A) and methanol (B), both with 5 mM ammonium formate, at a flow rate of 0.35 mL min^−1^. The injection volume was 20 µL. The gradient started with 50% A for 0.5 min, then changed to 95% B from 0.5 to 3 min, held at 95% B for 4 min, and returned to the initial conditions between 7 and 7.1 min, maintaining until 9.0 min for equilibration.

MS/MS analysis was accomplished with an electrospray ionisation (ESI) source in negative ionisation mode using multiple reaction monitoring (MRM). The ESI settings were as follows: CUR: 25; IS: −4.5 kV; TEMP: 550 °C; GS1: 40; GS2: 60. The MRM transitions were *m*/*z* 319 → 275 (quantifier) and 319 → 160 (qualifier) for α/β-ZEL and *m*/*z* 321 → 277 (quantifier) and 321 → 303 (qualifier) for α/β-ZAL. The standard curve was quadratic from 25 to 1000 ng mL^−1^ (r = 0.9992 (α-ZEL); r = 0.9992 (β-ZEL); r = 0.9995 (α-ZAL); r = 0.9997 (β-ZAL)).

#### 4.5.3. Qualitative Analysis of Zearalenone Biodegradation and Biotransformation Derivatives

Chromatographic separation was performed using an ExionLC AC system (SCIEX, Framingham, MA, USA). A sample volume of 5 µL was injected onto a Kinetex C18 column (100 × 2 mm, particle size: 2.6 µm; Phenomenex, Torrance, CA, USA). The mobile phases were water (A) and acetonitrile (B), both with 0.1% formic acid. Separation was performed at a flow rate of 0.5 mL/min with a column temperature of 40 °C. The gradient was held at 2% A for 1 min, then increased to 98% B from 1 to 15 min, held at 98% B for 5 min, and returned to the initial conditions between 20 and 20.1 min, maintaining until 22 min for equilibration.

The mass spectrometry analysis was performed on a ZenoTOF 7600 system (SCIEX, Framingham, MA, USA) in Independent Data Acquisition (IDA) mode. Spectra were acquired using an electrospray ionisation (ESI) source operating in positive or negative ion modes and collision-induced dissociation (CID) fragmentation to identify compounds. The ESI source conditions were set as follows for positive and negative polarity, respectively: spray voltage (5/–4.5 kV), curtain gas (35/35 psi), CAD gas (7/7 psi), temperature (550/550 °C), ion source gas 1 (nebuliser gas, 45/45 psi), ion source gas 2 (turbo gas, 60/60 psi), declustering potential (80/−80), collision energy (40/−40 V), and collision energy spread (15/−15 V). The scanning range was 100–840 *m*/*z* for precursor ions search (TOF MS) and 30–840 *m/z* mass spectra collection (TOF MS/MS). The criteria for IDA mode were as follows: maximum candidate ion (10), intensity threshold exceeds 200 counts, dynamic background subtract (ON), exclude former candidate ions (OFF), dynamic collision energy for MS/MS (OFF), exclude isotope (ON), and mass tolerance +/− (5 ppm).

Raw data were acquired using SCIEX OS 3.0 software (SCIEX, Framingham, MA, USA). Chromatograms of two samples were analysed, i.e., a set of the biotic control (without ZEN addition) and the corresponding tested sample (with ZEN addition), to find unique or intense peaks present in the sample with ZEN. Subsequent data processing employed the integrated Molecule Profiler software v. 1.3 within SCIEX OS (SCIEX, Framingham, MA, USA), which facilitates structural elucidation by automatically interpreting MS/MS fragment ions, enabling the identification of putative metabolites. A comprehensive list of 177 possible ZEN transformations was compiled to aid in this process, encompassing 80 pre-existing and 97 custom-designed derivatives. This list encompassed both phase I and II metabolic reactions, providing a thorough framework for metabolite identification.

### 4.6. Data Analysis

All experiments were conducted in triplicate, and sample variability was expressed as standard deviations (±SD). A Shapiro–Wilk test was used to assess the normality of the data distribution. Statistical significance was determined using one-way ANOVA followed by a Tukey’s post hoc test for normally distributed data. For non-normally distributed data, a Kruskal–Wallis test, a non-parametric equivalent of one-way ANOVA, was used, with multiple comparisons of the *p*-values. Statistical analyses were performed using GraphPad Prism v. 10.4.1 (GraphPad Software, San Diego, CA, USA).

## 5. Conclusions

This study demonstrated that *M. anisopliae* not only biotransformed ZEN to zearalenols and zearalanols but also exhibited the remarkable capability of cleaving the lactone ring structure of ZEN. This crucial step is often associated with a more complete detoxification of this mycotoxin, highlighting the fungus’s potential for bioremediation. Furthermore, our findings revealed the involvement of CYP450 enzymes in the ZEN degradation by this fungus. Future research should aim to identify and characterise additional enzymes to gain a more comprehensive understanding of the ZEN degradation pathway employed by *M. anisopliae*. This knowledge will facilitate the development of targeted strategies for enhancing ZEN biodegradation and harnessing the full bioremediation potential of this fungus. Ultimately, this research paves the way for exploring the potential of *M. anisopliae* as a biocontrol agent for mitigating ZEN contamination in agricultural environments, contributing to safer food and feed production.

## Figures and Tables

**Table 1:** Zearalenone removal by *M. anisopliae* at the initial concentration of 1 mg L^−1^.

Incubation Time [Days]	ZEN Residue [mg L^−1^]	
	AC	ZEN
1	0.99 ± 0.03	0.63 ± 0.01
2	0.99 ± 0.01	0.53 ± 0.00
3	0.99 ± 0.02	0.47 ± 0.00
4	0.99 ± 0.02	0.37 ± 0.00
5	0.99 ± 0.02	0.21 ± 0.01
6	0.99 ± 0.01	0.18 ± 0.00
7	0.98 ± 0.02	0.11 ± 0.00
10	0.98 ± 0.03	0.11 ± 0.00
14	0.99 ± 0.02	0.09 ± 0.01

**Table 2:** Metabolites of zearalenone generated in phase I and phase II, detected in ESI(-), as [M-H]^−^ ions.

ID	Chemical Formula	RT [min]	Neutral Mass	Theoretical *m*/*z*	Measured *m*/*z*	ppm	Transformation	Fragments, CID (Maximum 6 Most Intensive)
1	C_18_H_22_O_5_	12.35	318.146	317.1394	317.1387	−2.4	parent, zearalenone	317.1 (100), 273.1 (20), 187.0 (20), 175.0 (53), 160.0 (26), 131.1 (63)
2	C_18_H_24_O_5_	11.71	320.1618	319.1551	319.1545	−1.9	+ (H2), α-zearalenol	319.2 (100), 275.2 (24), 188.0 (9), 174.0 (14), 160.0 (20), 130.0 (18)
3	C_18_H_24_O_5_	10.99	320.1608	319.1551	319.1549	−0.6	+ (H2), β-zearalenol	319.2 (100), 301.1 (7), 275.2 (21), 188.0 (7), 174.0 (11), 160.0 (17)
4	C_18_H_26_O_5_	11.56	322.177	321.1707	321.1697	−3.3	+ (H4), α-zearalanol	321.2 (100), 303.2 (19), 277.2 (46), 205.1 (6), 191.1 (6), 161.1 (5)
5	C_18_H_26_O_5_	10.78	322.1769	321.1707	321.1696	−3.6	+ (H4), β-zearalenol	321.2 (100), 303.2 (19), 293.2 (3), 277.2 (46), 205.1 (6), 161.1 (5)
6	C_18_H_30_O_5_	13.14	326.2078	325.202	325.2006	−4.6	+ (H8)	181.1 (54), 165.1 (15), 143.1 (100), 138.1 (24), 121.1 (6), 97.1 (12)
7	C_15_H_24_O_3_	13.08	252.1717	251.1653	251.1644	−3.3	+ (H2) − (C3O2)	251.2 (54), 207.1 (54), 205.1 (26), 193.1 (67), 179.1 (100), 122.0 (54)
8	C_18_H_30_O_5_	9.66	326.2082	325.202	325.2009	−3.5	+ (H8)	181.1 (34), 165.1 (25), 143.1 (100), 138.1 (34), 121.1 (5), 97.1 (10)
9	C_15_H_24_O_3_	11.73	252.1714	251.1653	251.1641	−4.7	+ (H2) − (C3O2)	233.2 (36), 207.1 (73), 179.1 (100), 122.0 (90), 109.0 (82), 73.0 (46)
10	C_18_H_22_O_9_	5.96	382.1252	381.1191	381.1179	−3.2	+ (O4)	177.1 (100), 167.0 (51), 159.0 (90), 153.1 (75), 147.0 (72), 123.0 (48)
11	C_18_H_22_O_9_	5.66	382.1253	381.1191	381.118	−2.8	+ (O4)	177.1 (100), 159.0 (55), 153.1 (64), 147.0 (38), 123.0 (29), 99.0 (30)
12	C_19_H_24_O_5_	10.39	332.1614	331.1551	331.1541	−2.9	+ (C1H2)	331.2 (72), 207.1 (100), 165.1 (65), 151.0 (36), 139.0 (19), 115.0 (26)
13	C_19_H_26_O_4_	11.97	318.1819	317.1758	317.1747	−3.7	+ (C1H4) − (O1)	317.2 (76), 191.1 (100), 177.1 (27), 166.1 (28), 162.0 (16), 112.0 (14)
14	C_15_H_22_O_3_	13.13	250.1557	249.1496	249.1485	−4.6	– (C3O2)	249.1 (64), 205.2 (34), 203.1 (100), 191.1 (13), 177.1 (14)
15	C_15_H_22_O_3_	9.57	250.1563	249.1496	249.149	−2.4	− (C3O2)	213.1 (10), 141.1 (100), 139.1 (49), 109.1 (24), 59.0 (24), 57.0 (18)
16	C_18_H_24_O_4_	13.75	304.1663	303.1602	303.159	−3.9	+(H2) − (O1)	189.1 (6), 165.1 (12), 161.1 (23), 83.0 (6), 81.0 (100), 57.0 (7)
17	C_16_H_20_O_5_	10.23	292.1301	291.1238	291.1228	−3.5	− (C2H2)	163.1 (20), 125.0 (100), 85.0 (29), 65.0 (44)
18	C_14_H_14_O_6_	7.21	278.0788	277.0718	277.0715	−0.9	− (C4H8) + (O1)	175.0 (37), 149.1 (87), 133.0 (41), 121.1 (45), 109.0 (100), 107.1 (77)
19	C_17_H_24_O_3_	11.71	276.1725	275.1653	275.1652	−0.3	+ (H2) − (C1O2)	275.2 (80), 187.1 (100), 109.0 (78), 59.0 (98)
20	C_15_H_20_O_3_	11.87	248.1404	247.134	247.1331	−3.3	– (C3H2O3)	203.1 (100), 133.1 (93), 123.1 (64)
21	C_15_H_20_O_5_	7.36	280.1299	279.1238	279.1226	−4.1	– (C3H2)	190.1 (34), 163.1 (100), 107.0 (52), 79.1 (37)
22	C_12_H_14_O_4_	8.33	222.089	221.0819	221.0817	−1.1	– (C6H8O1)	179.1 (8), 135.0 (23), 107.1 (100), 105.1 (10), 91.1 (88), 65.0 (8)
23	C_8_H_8_O_3_	9.29	152.047	151.0401	151.0397	−2.3	– (C10H14O2)	107.1 (100), 106.0 (19), 92.0 (17)
24	C_7_H_8_O_3_	6.87	140.0468	139.0401	139.0396	−3.6	– (C11H14O2)	95.0 (100), 69.0 (34)
25	C_6_H_6_O_3_	5.37	126.0317	125.0244	125.0245	0.3	– (C12H16O2)	83.0 (21), 81.0 (100), 65.0 (7), 63.0 (8)

**Table 3:** List of primers used in the present study for RT-qPCR assay.

Gene Name	Primer Sequence (5′-3′)
*cyp53A25*	Forward: ACCATGCATCACTCCAAGGA Reverse: TCTCCATCTCTGCCACGTTT
*cyp55A20*	Forward: CTCCAAGGCCGATGTTGTTC Reverse: CCCCGATTTCAATGTCCGTC
*cyp55A19*	Forward: CAAGTTGCCACCCGATCAAA Reverse: AAGCAGGAGGAAGGCAATCT
*cyp505A21*	Forward: TGTTGACAGCACGCTAGAGA Reverse: TGGCCTCTTCGACATCTTGT
*cyp505M1*	Forward: TACCATCTCGTCCTCTTCGC Reverse: ACCGTGTCGAATTCCTCACT
*ubi*	Forward: AAGGGCGACATCAAGAAGGA Reverse: CGTTGACACCCATGACGTAC

## Data Availability

The raw MS data presented in the study are openly available in the University of Lodz Repository at http://hdl.handle.net/11089/54873 (accessed on 7 March 2025).
